# Phylogeny and Metabolic Potential of New Giant Sulfur Bacteria of the Family *Beggiatoaceae* from Coastal-Marine Sulfur Mats of the White Sea †

**DOI:** 10.3390/ijms25116028

**Published:** 2024-05-30

**Authors:** Nikolai V. Ravin, Tatyana S. Rudenko, Alexey V. Beletsky, Dmitry D. Smolyakov, Andrey V. Mardanov, Margarita Yu. Grabovich, Maria S. Muntyan

**Affiliations:** 1 Institute of Bioengineering, Research Center of Biotechnology, Russian Academy of Sciences, Leninsky Prospect, 33-2, 119071 Moscow, Russia; nravin@mail.ru (N.V.R.); mortu@yandex.ru (A.V.B.); mardanov@biengi.ac.ru (A.V.M.); 2 Department of Biochemistry and Cell Physiology, Voronezh State University, Universitetskaya pl., 1, 394018 Voronezh, Russia; ipigun6292@gmail.com (T.S.R.); songolifreya@mail.ru (D.D.S.); 3 Belozersky Institute of Physico-Chemical Biology, Lomonosov Moscow State University, Leninskie Gory, 119991 Moscow, Russia

**Keywords:** *Beggiatoaceae*, filamentous colorless sulfur bacteria, phylogeny, metagenome-assembled genome, ‘*Candidatus* Albibeggiatoa psychrophila’ gen. nov., sp. nov., ‘*Candidatus* Parabeggiatoa’, ‘*Candidatus* Parabeggiatoa communis’

## Abstract

The family *Beggiatoaceae* is currently represented by 25 genera in the Genome Taxonomy Database, of which only 6 have a definite taxonomic status. Two metagenome-assembled genomes (MAGs), WS_Bin1 and WS_Bin3, were assembled from metagenomes of the sulfur mats coating laminaria remnants in the White Sea. Using the obtained MAGs, we first applied phylogenetic analysis based on whole-genome sequences to address the systematics of *Beggiatoaceae*, which clarify the taxonomy of this family. According to the average nucleotide identity (ANI) and average amino acid identity (AAI) values, MAG WS_Bin3 was assigned to a new genus and a new species in the family *Beggiatoaceae*, namely, ‘*Candidatus* Albibeggiatoa psychrophila’ gen. nov., sp. nov., thus providing the revised taxonomic status of the candidate genus ‘BB20’. Analysis of 16S rRNA gene homology allowed us to identify MAG WS_Bin1 as the only currently described species of the genus ‘*Candidatus* Parabeggiatoa’, namely, ‘*Candidatus* Parabeggiatoa communis’, and consequently assign the candidate genus ‘UBA10656’, including four new species, to the genus ‘*Ca*. Parabeggiatoa’. Using comparative whole-genome analysis of the members of the genera ‘*Candidatus* Albibeggiatoa’ and ‘*Ca*. Parabeggiatoa’, we expanded information on the central pathways of carbon, sulfur and nitrogen metabolism in the family *Beggiatoaceae*.

## 1. Introduction

Colorless sulfur-oxidizing bacteria of the family *Beggiatoaceae*, classified as *Gammaproteobacteria* in the phylum *Pseudomonadota*, are among the largest prokaryotes. Members of the family are characterized by diverse morphology, ranging from filamentous trichome forms, varying from 1 to 200 µm, to spherical forms with sizes up to 750 µm. Some of the filamentous trichome forms are motile by gliding [1,2,3,4,5,6]. Representatives of *Beggiatoaceae* are inhabitants of sulfur biotopes and are able to use reduced sulfur compounds as electron donors for energy metabolism. In this case, their oxidation is accompanied by the accumulation of sulfur globules in invaginates of the cytoplasmic membrane and sulfates [5,6,7,8,9,10,11].

Despite more than a century and a half of research on *Beggiatoaceae*, there is only a fragmentary insight into the metabolism of the members of this group. Their taxonomy has undergone many changes during the entire period of studies, but has not yet been fully elaborated. The Bergey’s Manual, 1948 edition, contained four genera in the family: *Beggiatoa*, ‘T*hiospirillopsis*’, *Thioploca* and *Thiothrix* [12]. At that time, the only criterion used to assign cultures to *Beggiatoaceae* was trichome diameter. Half a century later, the taxonomy of the group changed significantly, and the genera *Thiothrix*, *Achromatium*, *Beggiatoa*, *Leucothrix*, *Thiobacterium*, *Thiomargarita* and *Thioploca* were combined into the family *Thiotrichaceae* [13]. However, this classification turned out to be erroneous because, according to the Genome Taxonomy Database (GDTB), the genera *Beggiatoa*, *Thiomargarita* and *Thioploca* constitute a separate phylogenetic branch distinct from the type genus *Thiothrix*, which, together with the genus *Leucothrix*, is now assigned to the family *Thiotrichaceae* (https://gtdb.ecogenomic.org/tree?r=o__Thiotrichales, accessed on 24 April 2024).

In 2011, based on trichome morphology and phylogenetic analysis using 16S rRNA, Salman et al. described seven candidate genera including vacuolated *Beggiatoa*-like filaments within the family *Beggiatoaceae*: ‘*Ca*. Marithioploca’, ‘*Ca*. Maribeggiatoa’, ‘*Ca*. Marithrix’, ‘*Ca*. Isobeggiatoa’, ‘*Ca*. Parabeggiatoa’, ‘*Ca*. Thiopilula’ and ‘*Ca*. Thiophysa’. They were shown to form a common monophyletic branch in class *Gammaproteobateria* together with the valid genera *Beggiatoa*, *Thiomargarita* and *Thioploca* [2]. At the same time, the candidate genera ‘*Ca*. Allobeggiatoa’ and ‘*Ca*. Halobeggiatoa’ were described [3,4]. Two additional candidate genera, ‘C*andidatus* Thiolava veneris’ and ‘C*andidatus* Venteria ishoeyi’, belonging to the family *Beggiatoaceae*, were described in 2017 [14,15]. In 2019, another genus, *Thioflexithrix,* with the type species *Thioflexithrix psekupsensis* represented by pure culture, was validated within the family *Beggiatoaceae* [6].

Accordingly, the family *Beggiatoaceae* has expanded markedly over the past seven decades, but so far pure cultures have been isolated only for the following three species: *Beggiatoa alba* and *Beggiatoa leptomitoformis* within the genus *Beggiatoa*, and *Thioflexithrix psekupsensis* within the genus *Thioflexithrix*. Consequently, the overwhelming majority of representatives of *Beggiatoaceae* currently have *Candidatus* status. The lack of pure cultures noticeably complicates the study on the physiology of the group, but, as can be assumed, has a lesser impact on taxonomy, given the impressive number of emerging genome sequences assembled from metagenomes and the phylogenome approach to prokaryote nomenclature that uses GDTB [16,17].

Phylogenetic data for nearly all genera of the family are not available because almost all descriptions predate the emergence of molecular phylogeny, and, with the exception of the earliest, are based solely on relatedness determined by 16S rRNA. Presently, according to GDTB, the family *Beggiatoaceae* comprises 25 genera that include 64 genome sequences (https://gtdb.ecogenomic.org/tree?r=o__Thiotrichales, accessed on 24 April 2024), while the LPSN contains 19 genera in the family *Beggiatoaceae* (https://lpsn.dsmz.de/family/beggiatoaceae, accessed on 28 May 2024 [18]). It is worth noting that, among them, only five genera are identified as those previously described; these are *Beggiatoa*, *Thioploca*, *Thiomargarita*, *Thioflexithrix* and ‘*Ca*. Marithrix’. Most likely, this is due to the fact that the 16S rRNA gene, which is key in resolving the argument regarding the taxonomy of both pure and enrichment cultures of the family, is lacking in most of the genome sequences obtained from metagenomic data. Presumably, among the unidentified genomes may be genomes belonging to representatives of the previously described genera that have only 16S rRNA gene sequences in GeneBank, and have no assembled whole genomes. The current state of taxonomy requires clarification of the data in order to identify previously described genera as well as new taxa.

In this study, we report the assembly of two metagenome-assembled genomes (MAGs), WS_Bin1 and WS_Bin3, from the metagenomes of bacterial sulfur mats formed on laminaria remnants at a depth of 15–18 m in the White Sea. We assigned MAG WS_Bin3 to a new genus named ‘*Ca*. Albibeggiatoa psychrophila’ gen. nov., sp. nov. within the family *Beggiatoaceae*. We assigned MAG WS_Bin1 to the species ‘*Ca*. Parabeggiatoa communis’, previously described by Salman et al. within the genus ‘*Ca*. Parabeggiatoa’, described by the same authors [2]. In that way, we identified two candidate genera, ‘BB20’ and ‘UBA10656’, as ‘*Ca*. Albibeggiatoa’ gen. nov. and ‘*Ca*. Parabeggiatoa’, respectively, which, according to GTDB, are genera within the family *Beggiatoaceae*. Analysis of the obtained genome sequences can help to broaden the overall insight into the metabolism of the genera ‘*Ca*. Parabeggiatoa’ and ‘*Ca*. Albibeggiatoa’, and, accordingly, the family *Beggiatoaceae* as a whole.

## 2. Results and Discussion

### 2.1. Biotop Features

**Figure 1:**
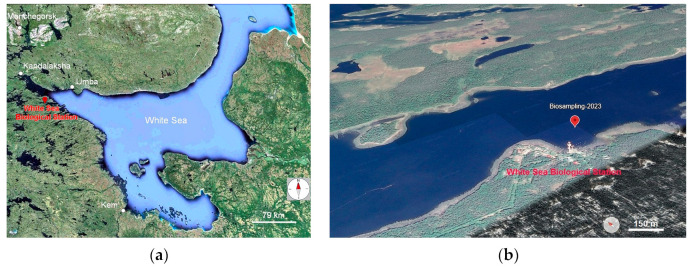
Geographical location of the fieldwork and biosampling site: (**a**) the Nikolai A. Pertsov White Sea Biological Station of the Biology Faculty of Lomonosov Moscow State University on the shore of the White Sea (red sign, Primorskiy settlement, Republic of Karelia, Russia). (**b**) The site of biosampling in the sea bay at the “Laminaria dump” (red sign) near the marine pier, which is visible on the map in the center of the station as a white construction on the edge of the shore, protruding into the bay.

The biotope, where the search for sulfur bacteria of the family *Beggiatoaceae* was performed, is a coastal–marine zone in the area of the N.A. Pertsov White Sea Biological Station of the Biological Faculty of Lomonosov Moscow State University (Primorsky settlement, Republic of Karelia, Russia) (Figure 1a). The marine bio-sampling site is located in the White Sea bay called “Laminaria dump”, within 100 m from the sea pier of the N.A. Pertsov White Sea Biological Station (Figure 1b). Here, at a depth of 15–18 m, layers of decomposing laminaria thalloms accumulate, which are brought here by natural marine underwater currents. On the surface of the decomposing laminaria, intense whitish sulfur mats form. The seabed of the “Laminaria dump” is heavily covered with silt and saturated with decomposing organics. Hydrogen sulfide is intensively formed here. The biotope is characterized by the following indicators: NaCl concentration is 20 g/L, oxygen concentration in the area of mat formation is 0.5–1 mg/L, sulfide concentration is 0.13 mM, seawater temperature is kept constant at +10 ± 4 °C, and the pH is 7.5–8.0.

**Figure 2:**
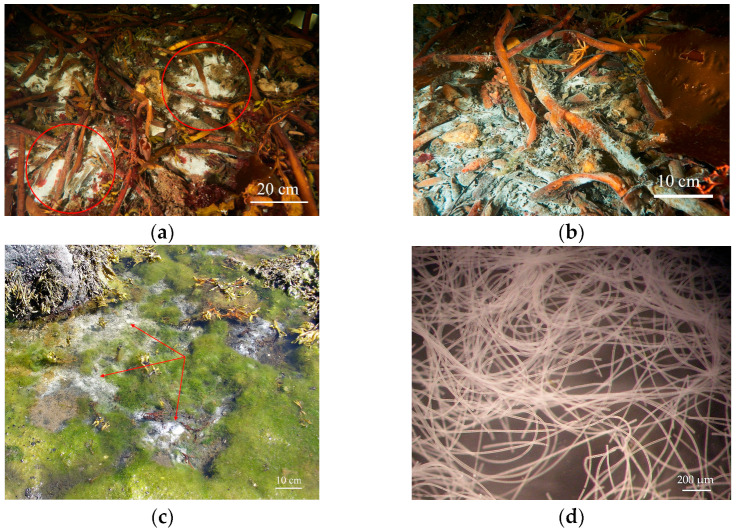
General view of bacterial sulfur mats visible as rough whitish coating composed of the representatives of the family *Beggiatoaceae*: (**a**,**b**) on the decaying laminaria thalomes at the marine bottom of the “Laminaria dump” in the White Sea (underwater photography)—(**a**) scant whitish mats circled in red; (**b**) intense whitish mats; (**c**) the intertindal zone of the White Sea (mats selectively shown by red arrows, binocular microscope Nikon SMZ445). (**d**) Close-up of filaments collected from sulfur mats in the intertindal zone. Photographs (**c**,**d**) provided by M.Yu. Grabovich.

For metagenomic analysis, samples of bacterial sulfur mats formed on laminaria remnants were collected in the sea bay “Laminaria dump” of the White Sea 100 m northeast of the marine pier of the N.A. Pertsov White Sea Biological Station (66°34′16.94″ N, 33°06′23.44″ E) (Figure 2a,b) at a depth of 15 m, where the sea water temperature was 14 °C, on 7 August 2023. It should be noted that sulfur mats containing giant filaments of colorless sulfur bacteria of the family *Beggiatoaceae* were also found in the intertidal zone of the White Sea (Figure 2c,d).

### 2.2. Morphology of Filamentous Colorless Sulfur Bacteria from Sulfur Mats

**Figure 3:**
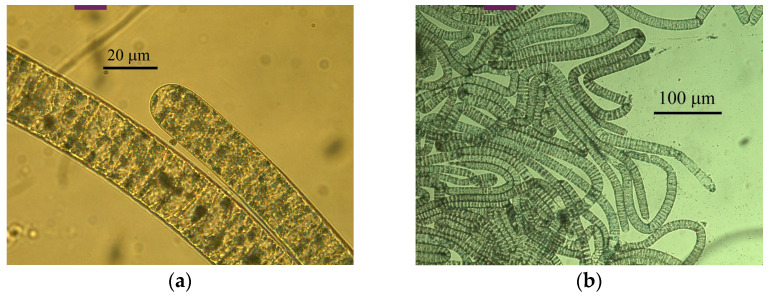

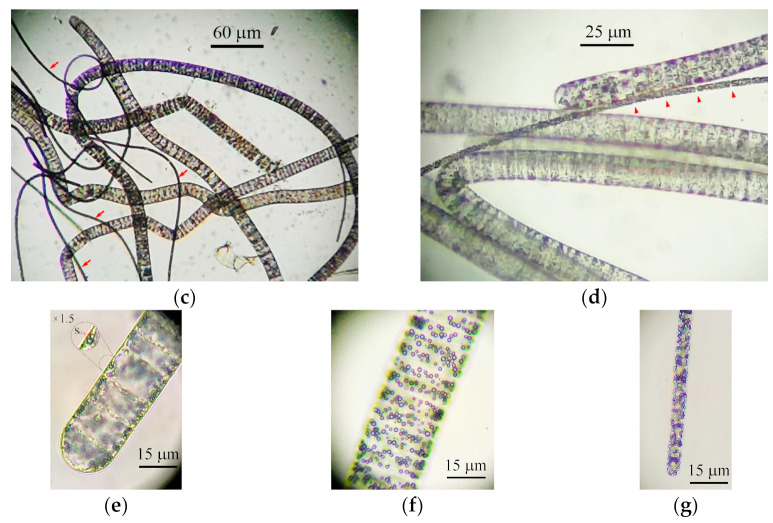
Microscopy views of filaments from bacterial sulfur mats collected in the sea bay “Laminaria dump”, the White Sea. Cell morphology of the (**a**–**f**) giant and (**c**,**d**,**g**) medium-sized (shown by red arrows) bacteria. (**a**,**e**,**f**) Disk-shaped cells of the giant bacteria and (**d**) cylindrical cells of the medium-sized bacteria (red arrow heads show the boundaries between neighboring cells). (**e**–**g**) Multiple highly light-refracting rounded inclusions, which in sulfur-oxidizing bacteria are represented by globules of elemental sulfur (**e**,**f**) in giant bacterial cells, and (**g**) in medium-sized bacterial cells. (**e**) Globules of elemental sulfur accumulate in giant bacteria; globules are selectively shown in 1.5× magnification (red arrows, inset, top left corner). (**a**) Phase-contrast microscopy, (**b**–**g**) light microscopy. Photographs provided by (**a**,**b**) M.Yu. Grabovich and (**c**–**g**) M.S. Muntyan.

Microscopy analysis of colorless filamentous sulfur bacteria from the sampled bacterial sulfur mats revealed two types of filaments of large and medium sizes with morphologies characteristic of representatives of the family *Beggiatoaceae* (Figure 3a–d). Filaments of the first type consisted of large colorless disc-shaped cells of 18.0–20.0 × 8.0–10.0 μm in size with rounded ends, which formed trichomes up to 900 μm long (Figure 3a,b,e,f). The cells contained intracellular inclusions of elemental sulfur (Figure 3e,f) and presumably vacuoles, similar to the cells of ‘*Ca*. Parabeggiatoa’ and ‘*Ca*. Isobeggiatoa divolgata’ [1,2]. The filaments were motile by gliding.

The cells of the medium-sized filaments were of 1.0–2.5 × 3.5–4.0 μm in size, cylindrical in shape with rounded ends, and formed trichomes up to 300 μm long (Figure 3c,d). They did not contain vacuoles but had intracellular sulfur globules (Figure 3g). The trichomes were motile by gliding.

### 2.3. Assembly of Beggiatoaceae MAGs

Two MAGs, MAG WS_Bin1 and MAG WS_Bin3, from representatives of the family *Beggiatoaceae* were assembled from the obtained metagenomes (Table 1). MAG WS_Bin1 was assembled from 1575 contigs (N50 size was 6604 bp) with a total length of 8,639,748 bp. CheckM2 estimated the completeness of this MAG as 91.63% with a possible contamination of 3.05%. Genome annotation identified a 16S rRNA gene, 45 tRNA genes and 6942 potential protein-coding genes. The GC of the genome is 41.0%.

MAG WS_Bin3 was assembled from 708 contigs (N50 size was 6229 bp) with a total length of 3,641,550 bp. CheckM2 estimated genome assembly completeness of 95.95% with possible contamination of 1.29%. Genome annotation identified a 16S rRNA gene, 33 tRNA genes and 2800 potential protein-coding genes. The GC of the genome is 38.0%.

The general characteristics of the genomes are summarized in Table 1.

### 2.4. Phylogenetic Analysis of the MAG Beggiatoaceae sp. WS_Bin1

Analysis of relatedness using the 16S rRNA gene showed that the MAG *Beggiatoaceae* sp. WS_Bin1 is 99.87% homologous with the species ‘*Ca*. Parabeggiatoa communis’ attributed to the genus ‘*Ca*. Parabeggiatoa’. No genome sequence data for this genus are available as yet. In 2011, Salman et al. described this genus based only on the identity of 16S rRNA genes [2]. According to 16S rRNA gene homology, the obtained MAG WS_Bin1 can be attributed to the previously described species ‘*Ca*. Parabeggiatoa communis’ within the genus ‘*Ca*. Parabeggiatoa’.

A GTDB search attributed WS_Bin1 to the family *Beggiatoaceae* within the non-identified genus ‘UBA10656’, which includes the following six genomes: MAG 4572_84 [19], MAG B3_G6, MAG B38_G9, MAG B2_G13, MAG B37_G9 and MAG B5_G6 [20].

**Figure 4:**
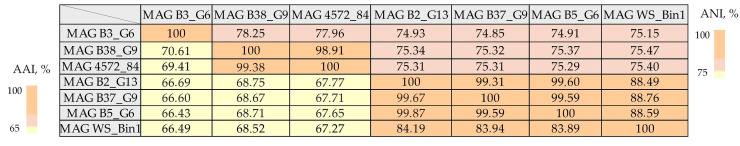
Heatmap of pairwise AAI and ANI values (%) for ‘*Ca*. Parabeggiatoa’: MAG WS_Bin1 (GCA_035871815.1); MAG B3_G6 (GCA_003646175.1); MAG 4572_84 (GCA_002085445.1); MAG B38_G9 (GCA_003646135.1); MAG B5_G6 (GCA_003645185.1); MAG B37_G9 (GCA_003645245.1); MAG B2_G13 (GCA_003645315.1).

The pairwise amino acid identity (AAI) values for MAG WS_Bin1 and other genomes included in the analysis are in the range of 66.49–83.89% (above the 65% threshold adopted for genus differentiation) (Figure 4), which allows the unambiguous description of the group ‘UBA10656’ and the obtained new MAG WS_Bin1 as the genus ‘*Ca*. Parabeggiatoa’, described by Salman et al. in 2011 [2,21].

The pairwise nucleotide identity (ANI) values estimated within the species in the genus ‘*Ca*. Parabeggiatoa’ are in the range of 75.15–88.76% (Figure 4). Among the seven MAGs in the group ‘UBA10656’, the data obtained allow us to identify four independent species within the genus ‘*Ca*. Parabeggiatoa’: ‘*Ca*. Parabeggiatoa’ sp. nov. with MAG B3_G6, ‘*Ca*. Parabeggiatoa’ sp. nov. with two MAGs 4572_84 and B38_G9, and ‘*Ca*. Parabeggiatoa’ sp. nov. with three MAGs B5_G6, B37_G9 and B2_G13, as well as the MAG WS_Bin1, which we obtained and assigned to ‘*Ca*. Parabeggiatoa communis’. In the present study, we did not introduce species names for the identified genomes, and therefore use the following designations: ‘*Ca*. Parabeggiatoa’ sp. nov. 1 with MAG B3_G6; ‘*Ca*. Parabeggiatoa’ sp. nov. 2, comprising two MAGs 4572_84 and B38_G9; ‘*Ca*. Parabeggiatoa’ sp. nov. 3, comprising three MAGs B5_G6, B37_G9 and B2_G13.

**Figure 5:**
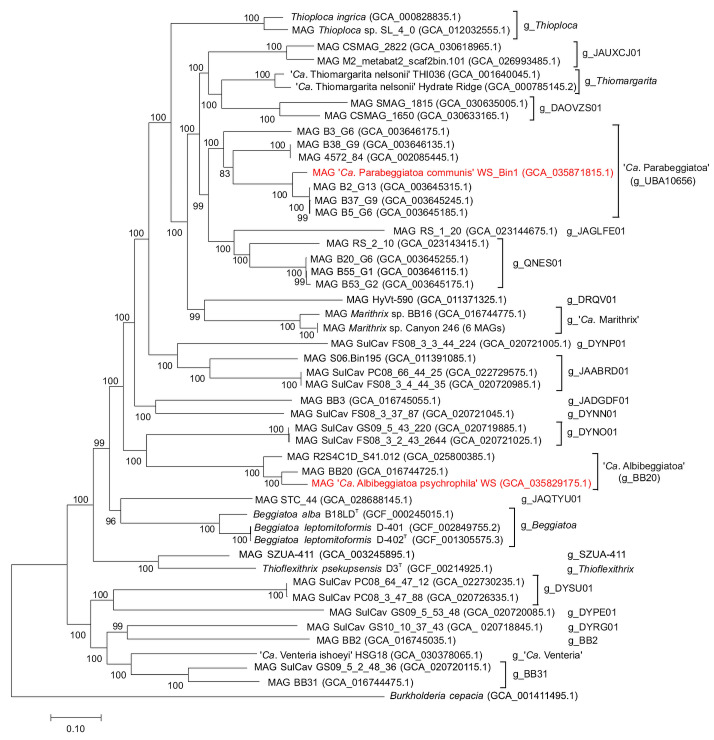
Phylogenetic tree based on genome sequences of the family *Beggiatoaceae*. The genome positions were determined by the maximum-likelihood method using concatenated sequences and 120 conserved marker genes. The two MAGs, WS_Bin1 and WS_Bin3, assembled in this study, are shown in red font.

Genome positions on the phylogenetic tree were determined basing on concatenated sequences and 120 conserved marker genes (Figure 5).

### 2.5. Phylogenetic Analysis of the MAG Beggiatoaceae sp. WS_Bin3

BLAST search using 16S rRNA gene sequence of the MAG sequence of *Beggiatoaceae* sp. WS_Bin3 showed 97.24% homology and the species difference with the marine strain *Beggiatoa* sp. 35Flor, which was previously attributed to the genus *Beggiatoa* based on its similar morphology and physiology. The 16S rRNA gene homology of *Beggiatoa* sp. 35Flor with the canonical representatives of the genus *Beggiatoa* is 85.95% and is below the limit of difference between the two genera. However, due to the lack of pure culture and genome sequence, the strain *Beggiatoa* sp. 35Flor has shown no definite taxonomic status to date.

According to GTDB, MAG WS_Bin3 is attributed to the family *Beggiatoaceae* within the candidate genus ‘BB20’, represented by a single genome, MAG BB20. The pairwise ANI value of the two genomes is 84.23%. The obtained value allows us to assign these genomes to two different species within the new genus ‘*Ca*. Albibeggiatoa’ gen. nov. with the proposed names ‘*Ca*. Albibeggiatoa psychrophila’ gen. nov., sp. nov. WS and ‘*Ca*. Albibeggiatoa’ sp. nov. BB20, thus providing the revised taxonomic status of the candidate genus ‘BB20’—‘*Ca*. Albibeggiatoa’ gen. nov.

Complementary BLAST searching against the sequence of the gene *tilS* from MAG WS_Bin3 revealed the genome sequence of *Thiotrichaceae* sp. R2S4C1D_S41.012 [22]. The degree of homology of gene *tilS* is indicative of the phylogeny of the genus *Thiothrix*, but the analysis performed is tentative for the genus *Beggiatoaceae* and was fulfilled to search for new genome sequences [23]. ANI analysis of the detected genome R2S4C1D_S41.012 in relation to genomes of ‘*Ca*. Albibeggiatoa psychrophila’ and ‘*Ca*. Albibeggiatoa’ sp. nov. BB20 showed values of 79.9% and 84.23%, which allowed us to identify *Thiotrichaceae* sp. R2S4C1D_S41.012 as a new species within the genus ‘*Ca*. Albibeggiatoa’ sp. nov. NOAA.

Genome positions on the phylogenetic tree were determined based on concatenated sequences and 120 conserved marker genes (Figure 5).

The full-genome sequence of *Beggiatoa* sp. 35Flor was unavailable; therefore, identifying the taxonomic status of *Beggiatoa* sp. 35Flor was difficult. According to 16S rRNA gene homology, the strain was unambiguously attributed to the genus ‘*Ca*. Albibeggiatoa’. However, to define *Beggiatoa* sp. 35Flor as a new species within this genus or assign it to the existing species ‘*Ca*. Albibeggiatoa’ sp. nov. BB20 and ‘*Ca*. Albibeggiatoa’ sp. nov. NOAA proved to be impossible. Therefore, we suggest reclassifying this strain within a new genus with the proposed name ‘*Ca*. Albibeggiatoa’ sp. 35Flor comb. nov.

### 2.6. Genome Analysis of the Main Metabolic Pathways of Representatives of ‘Candidatus Parabeggiatoa’

A pure culture of ‘*Ca*. Parabeggiatoa’ has not yet been obtained, and all knowledge of its metabolism is derived from observations of bacteria in natural associations. All representatives of ‘*Ca*. Parabeggiatoa’, as the representatives of the family *Beggiatoaceae*, belong to sulfur-oxidizing bacteria. They form abundant sulfur mats in natural and anthropogenic water reservoirs with high hydrogen sulfide content [24].

**Figure 6:**
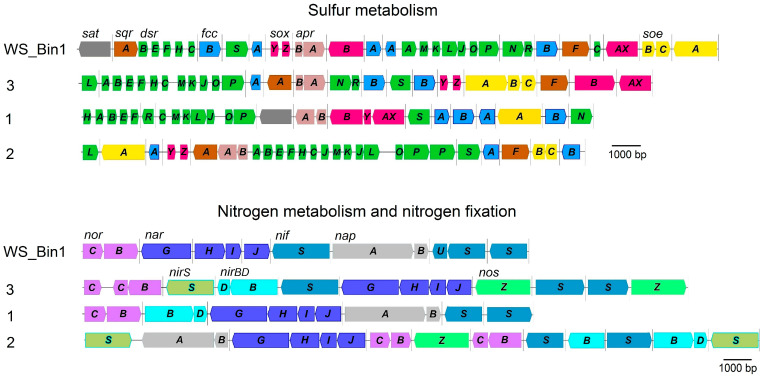
Order of genes encoding enzymes involved in dissimilatory sulfur and nitrogen metabolism and in nitrogen fixation in ‘*Ca*. Parabeggiatoa’. WS_Bin1, ‘*Ca*. Parabeggiatoa communis’; 3, ‘*Ca*. Parabeggiatoa’ sp. 3; 1, ‘*Ca*. Parabeggiatoa’ sp. 1; 2, ‘*Ca*. Parabeggiatoa’ sp. 2. *narG*, membrane-bound nitrate reductase; *nirS*, dissimilatory nitrite reductase; *norBC*, nitric oxide reductase; *nosZ*, nitrous oxide reductase; *napAB*, periplasmic nitrate reductase; *nirBD*, assimilatory nitrite reductase; *nif*-gene cluster, nitrogenase genes; *sqr*, sulfide:quinone oxidoreductase; *fccAB*, flavocytochrome sulfide dehydrogenase; *soxAXBYZ*, the branched Sox-system; rDsr, complex for sulfur oxidation *dsrABEFHNEMKLJONR*; *soeABC*, quinone-dependent sulfite dehydrogenase; *aprAB*, adenosine 5′-phosphosulfate reductase; *sat*, ATP-sulfurylase, dissimilatory-type. The “sulfur metabolism” panel and the “nitrogen metabolism and nitrogen fixation” panel used different color palettes to distinctively label genes of different systems, with no overlap between the two panels.

During the annotation of ‘*Ca*. Parabeggiatoa’ genomes, a set of systems involved in dissimilatory sulfur metabolism was found (Figure 6). This information is consistent with the few data on the ability to validly describe representatives of the family *Beggiatoaceae* to grow lithotrophically in the presence of reduced sulfur compounds [5,6,10,11]. This also indicates the active contribution of this group of bacteria to the cycling of sulfur compounds in nature, and their role in the maintenance of a stable state in ecosystems.

Thus, two systems of hydrogen sulfide oxidation were found in ‘*Ca*. Parabeggiatoa’: SQR (sulfide:quinone oxidoreductase) and FCSD (flavocytochrome sulfide dehydrogenase) (Figure 6, Table 2).

The genes *sqrF* and *sqrA* of two SQR types were found in the species of ‘*Ca*. Parabeggiatoa communis’ WS_Bin1, namely, the MAGs 4572_84 and B5_G6, with the exception of MAG B37_G9, in the genome of which only the gene *sqrA* was found. The genome of B3_G6 did not contain any genes encoding SQR.

FCSD genes are present in the genomes of all the representatives of the genus ‘*Ca*. Parabeggiatoa’. Thiosulfate oxidation is mediated by a branched Sox-system devoid of SoxCD proteins (Figure 6, Table 2). No individual genes encoding the second subunit of SoxAX proteins, SoxX, were found in the genomes, and the SoxAX protein is fused similarly to those in other representatives of the family *Beggiatoaceae* [5,6,25]. The genomes of all species contain genes of the Sox-complex, but a number of genes of this complex are absent in some MAGs. Gene *soxAX*, which initializes the first reaction of the multi-enzyme complex during thiosulfate oxidation, was not found in the genome of MAG 4572_84. Gene *soxZ*, whose product, together with SoxY, is involved in thiosulfate binding, was absent from B3_G6 genome. The gene encoding SoxB, which is involved in the hydrolysis reaction, was absent from the genome of MAG B2_G13 [26]. In general, it could be assumed that without these genes, bacteria were unable to oxidize thiosulfate; however, given the high relatedness of the genomes within the species (ANI > 99%), the described mosaic loss of individual genes may be the result of poor genome assembly.

Elemental sulfur, which is one of the products of the activity of the Sox-system and SQR/FCSD, is oxidized via the rDSR-complex in ‘*Ca*. Parabeggiatoa’. Despite the fact that the rDSR-complex consists of a set of proteins with complex functional dependence (*dsrABEFHNEMKLJONR*), the system under consideration is one of the most complete along with other systems of dissimilatory sulfur metabolism in the genus ‘*Ca*. Parabeggiatoa’ (Figure 6, Table 2).

Annotation of the genomes of ‘*Ca*. Parabeggiatoa’ revealed that the systems of sulfite oxidation are the most fragmented and variable in gene composition. Thus, genes encoding the system of indirect sulfite oxidation by APS-reductase and ATP-sulfurylase (*aprAB* and *sat*) were found in the genomes of two MAGs B2_G13 and B37_G9, and in the species ‘*Ca*. Parabeggiatoa’ sp. nov. 1. Genes of two systems *soeABC*, encoding the cytoplasmic membrane-bound sulfite:quinone oxidoreductase SoeABC complex, and *aprAB*/*sat*, were found in WS_Bin1, 4572_84 and B5_G6. Genes *soeC* and *sat*, related to the direct and indirect sulfite oxidation pathway, were not detected in MAG B2_G13, but the presence of gene *sat* in the related MAG B37_G9 suggests the loss of this gene during the assembly of genome B2_G13. Genes *aprAB* encoding APS-reductase, which catalyzes sulfite oxidation in ATP-sulfurylase-coupled catalysis, were found in all representatives of the species ‘*Ca*. Parabeggiatoa’ (Figure 6, Table 2).

The presence of intracellular vacuoles that presumably accumulate nitrate at high concentrations (up to 800 mM) is typical for marine filamentous giant sulfur bacteria [27,28,29,30,31]. Early studies with the enrichment culture of these bacteria testify that nitrate may be involved in nitrate reduction or denitrification processes, allowing sulfide oxidation at low oxygen concentrations [27,28,29,31]. The genome analysis of ‘*Ca*. Parabeggiatoa’ spp. revealed all genes corresponding to denitrification and dissimilatory nitrate reduction.

Complete denitrification genes (*narGHI*, *nirS*, *norBC* and *nosZ*) are present in the genomes of species ‘*Ca*. Parabeggiatoa’ sp. nov. 2 and ‘*Ca*. Parabeggiatoa’ sp. nov. 3. The genomes of B3_G6 and ‘*Ca*. Parabeggiatoa communis’ WS_Bin1 contain no genes *nirS*, encoding nitrite reductase, which catalyzes the conversion of NO_2_^−^ to NO, as well as no genes *nosZ*, encoding nitrous-oxide reductase, involved in the reduction of N_2_O to N_2_. Genes *napAB*, encoding periplasmic nitrate reductase of dissimilatory type, involved in nitrate reduction, and *nirBD* for nitrite reductase, catalyzing dissimilatory reduction of nitrite to ammonium, respectively, were disclosed in the genomes of species ‘*Ca*. Parabeggiatoa’ sp. nov. 1 and ‘*Ca*. Parabeggiatoa’ sp. nov. 2. Despite the presence of genes *nirBD*, no genes *napAB* were detected in the species ‘*Ca*. Parabeggiatoa’ sp. nov. 3, while during annotation, no genes *nirBD* were detected, although genes *napAB* were found in ‘*Ca*. Parabeggiatoa communis’ WS_Bin1. Molecular nitrogen fixation genes represented by the *nif*-cluster are absent from all representatives of the genus ‘*Ca*. Parabeggiatoa’ (Figure 6, Table 2).

All genes of the Calvin–Benson–Bassham cycle, including genes of the key enzymes, RuBisCO and phosphoribulokinase, were found in representatives of the genus *Ca*. Parabeggiatoa’. Genes of type II RuBisCO were found in the genomes of ‘*Ca*. Parabeggiatoa communis’ WS_Bin1 and genomes belonging to the species ‘*Ca*. Parabeggiatoa’ sp. nov. 3. Type IAq RuBisCO is encoded in the genomes of the species ‘*Ca*. Parabeggiatoa’ sp. nov. 1 and ‘*Ca*. Parabeggiatoa’ sp. nov. 2. Accordingly, the presence of a number of systems of dissimilatory sulfur metabolism, as well as Calvin–Benson–Bassham cycle genes, suggests the capacity of representatives of the genus ‘*Ca*. Parabeggiatoa’ for lithoautotrophy (Table 2).

Central carbon metabolism is represented by glycolysis and the Krebs cycle, but the gene *glk* for classic glucokinase is absent from ‘*Ca*. Parabeggiatoa communis’ WS_Bin1 and the genomes of the species ‘*Ca*. Parabeggiatoa’ sp. nov. 2. The gene *gpmI* for 2,3-bisphosphoglycerate-independent phosphoglycerate mutase is absent from all representatives. All Krebs cycle genes are represented in the genomes of ‘*Ca*. Parabeggiatoa’, but genes for key enzymes of the glyoxylate cycle, in particular malate synthase (*aceB*) and isocitrate lyase (*aceA*), are absent.

In the genomes of ‘*Ca*. Parabeggiatoa’, no genes for methylotrophic growth were detected; particularly, the genes for methanol dehydrogenase and C1-carbon assimilation cycles via ribulose monophosphate and serine cycles. The capacity for methylotrophic growth was shown for representatives of the genus closely related to *Beggiatoa* [32,33].

### 2.7. Genome Analysis of the Main Metabolic Pathways of Representatives of ‘Candidatus Albibeggiatoa’ gen. nov.

**Figure 7:**
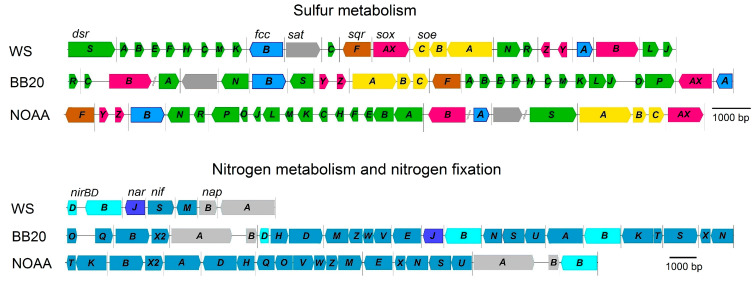
Order of genes encoding enzymes involved in dissimilatory sulfur and nitrogen metabolism and in nitrogen fixation in ‘*Ca*. Albibeggiatoa’. WS, ‘*Ca*. Albibeggiatoa psychrophila’; BB20 ‘*Ca*. Albibeggiatoa’ sp.; NOAA, ‘*Ca*. Albibeggiatoa’ sp. *narG*, Membrane-bound nitrate reductase; *nirS*, dissimilatory nitrite reductase; *napAB*, periplasmic nitrate reductase; *nirBD*, assimilatory nitrite reductase; *nif*-gene cluster, nitrogenase genes; *sqr*, sulfide:quinone oxidoreductase; *fccAB*, flavocytochrome sulfide dehydrogenase; *soxAXBYZ*, the branched Sox-system; rDsr, complex for sulfur oxidation *dsrABEFHNEMKLJONR*; *soeABC*, quinone-dependent sulfite dehydrogenase; *sat*, ATP-sulfurylase, dissimilatory-type. The same colors were used to denote gene systems with the same names as in Figure 6. The color palettes used are given in the legend to Figure 6.

Genome analysis of representatives of the genus ‘*Ca*. Albibeggiatoa’ gen. nov. showed the presence of a number of genes responsible for the dissimilatory metabolism of sulfur, typical for representatives of the family *Beggiatoaceae*. Genomes of ‘*Ca*. Albibeggiatoa psychrophila’ sp. nov. WS, ‘*Ca*. Albibeggiatoa’ sp. nov. BB20 and ‘*Ca*. Albibeggiatoa’ sp. nov. NOAA contain genes *sqrF*, encoding type VI SQR, and *fccAB*, encoding FCSD for hydrogen sulfide oxidation to elemental sulfur; genes *dsrABEFHNEMKLJONR*, encoding the rDSR-complex for sulfur oxidation to sulfite; genes *soeABC*, encoding the membrane-bound complex of cytoplasmic sulfite:quinone oxidoreductase SoeABC for direct sulfite oxidation to sulfate. Thiosulfate is oxidized by a branched Sox-system, the genes of which are present in three species. Similar to representatives of ‘*Ca*. Parabeggiatoa’, genes for the fused protein SoxAX were found in genomes of ‘*Ca*. Albibeggiatoa’. Gene *aprAB*, encoding APS-reductase for indirect sulfite oxidation, was not found, although gene *sat*, encoding ATP-sulfurylase, which is most likely involved in assimilatory sulfate reduction processes, was present (Figure 7, Table 2).

Nitrogen metabolism is represented by genes of dissimilatory nitrate reduction. Three genomes, namely, WS, BB20 and NOAA, contain genes *napAB*, encoding periplasmic nitrate reductase of the dissimilatory type, involved in nitrate reduction, and the gene *nirBD*, encoding nitrite reductase, catalyzing the reduction of nitrite to ammonium. All genes *nifASUVNWMT* responsible for the maturation of nitrogenase complex and genes *nifDHKI*, encoding catalytic subunits, were found in genomes of the MAGs BB20 and NOAA, while genes responsible for molecular nitrogen fixation are absent from the genome WS.

The genes for assimilatory nitrate reduction, the gene of the catalytic subunit of assimilatory nitrate reductase, involved in the reduction of nitrate to nitrite, and genes *nasBDE*, encoding assimilatory nitrite reductase, involved in nitrite assimilation, were found in the genomes of the three species. The genes responsible for amination, *glnB*, *gltBD* and *aspB*, with the exception of *aspB* not identified in the genome WS, were also annotated in the genomes (Figure 7, Table 2).

The autotrophic assimilation of carbon dioxide is realized via the Calvin–Benson–Bassham cycle, and the genes for the key enzymes RuBisCO and phosphoribulokinase (*prk*) are presented in the genomes of the three species. Type II RuBisCO was detected (Table 2).

All genes of the Krebs cycle, oxidative pentose–phosphate pathway and glyoxylate shunt were found in the genomes. The presence of all genes encoding the electron- transport chain indicates a respiratory type of metabolism.

Similarly to representatives of the genus ‘*Ca*. Parabeggiatoa’, no genes responsible for methylotrophic growth were found in genomes of ‘*Ca*. Albibeggiatoa’.

### 2.8. Description of New Genus and Species

#### 2.8.1. Description of ‘*Candidatus* Albibeggiatoa’ gen. nov.

*Albibeggiatoa* (Al.bi.beg.gi.a.to’a. L. masc. adj. albus, white; N.L. fem. n. *Beggiatoa*, a bacterial genus name: N.L. fem. n. *Albibeggiatoa*, a white *Beggiatoa*).

Genbank accession numbers of genome sequence of species: GCA_035829175.1, GCA_016744725.1, GCA_025800385.1.

#### 2.8.2. Description of ‘*Candidatus* Albibeggiatoa psychrophila’ sp. nov.

*psychrophila* (psy.chro’phi.la. Gr. masc. adj. psychros, cold; N.L. masc. adj. suff. philus, loving; N.L. fem. adj. psychrophila, preferring the cold).

Not cultivated. Morphologically represented by cylindrical Gram-negative cells 1.0–2.5 × 3.5–4.0 μm in diameter with rounded ends; the cells form filaments. Filaments are motile by gliding. Their metabolism is of respiratory type. The Krebs cycle and glyoxylate cycle may be involved in catabolic and anabolic processes. Microaerobes. They are capable of dissimilatory and assimilatory reduction of nitrate to ammonium. Facultative lithoautotrophs capable of fixing carbon dioxide in the Calvin–Benson–Bassham cycle with type II RuBisCO. During lithoautotrophic growth, they obtain energy by oxidation of reduced sulfur compounds accompanied by the formation of intracellular sulfur globules and sulfate; oxidation of elemental sulfur to sulfite occurs via the Dsr-system. Polyphosphate metabolism genes are encoded in their genomes.

Source: MAG WS_Bin3 was assembled from the metagenome of bacterial sulfur mats coating laminaria remnants in the sea bay “Laminaria dump” in the White Sea (Primorskiy settlement, Republic of Karelia, Russia).

GC fraction of genomic DNA (%): 38.0 (genome sequence).

GenBank accession number (whole genome assembly): GCA_035829175.1.

#### 2.8.3. Emended Description of ‘*Candidatus* Parabeggiatoa’ Salman et al., 2011 (Lists of names of Prokaryotic *Candidatus* Taxa 2020) [34]

*Parabeggiatoa* (Pa.ra.beg.gi.a’to.a. Gr. prep. *para* next to; N.L. fem. n. *Beggiatoa* a bacterial genus; N.L. fem. n. *Parabeggiatoa* a genus next to *Beggiatoa*).

Not cultivated. Morphologically represented by disk-shaped Gram-negative cells 20–40 µm in diameter with rounded ends, the cells form filaments. Filaments are motile by gliding. Have vacuoles. Able to accumulate nitrate in vacuoles and elemental sulfur as intracellular inclusions. Metabolism is of respiratory type. Participation of the Krebs cycle in catabolic and anabolic processes is possible. Microaerobes. They are capable of dissimilatory and assimilatory reduction of nitrate to ammonium. Facultative lithoautotrophs capable of fixing carbon dioxide in the Calvin–Benson–Bassham cycle with types IAq and II RuBisCO. During lithoautotrophic growth, they obtain energy by oxidation of reduced sulfur compounds accompanied by the formation of intracellular sulfur globules and sulfate; oxidation of elemental sulfur to sulfite occurs via the Dsr-system. Not capable of molecular nitrogen fixation. Polyphosphate metabolism genes are encoded in their genomes.

Genbank accession numbers of genome sequence of species: GCA_035871815.1, GCA_003646175.1, (GCA_003646135.1, GCA_002085445.1), (GCA_003645315.1, GCA_003645245.1, GCA_003645185.1).

## 3. Materials and Methods

### 3.1. Geography and Physicochemical Characteristics of Environmental Sampling Sites for Metagenomic Characterization of MAG Beggiatoaceae sp. WS_Bin1 and MAG Beggiatoaceae sp. WS_Bin3

The fieldwork was carried out during the summer seasons in different years by M.Yu. Grabovich and M.S. Muntyan at the base for the Research and Education Centre «Marine Biology, Oceanography and Geology» of the White Sea Biological Station after Nikolai A. Pertsov of the Biology Faculty of Lomonosov Moscow State University (Primorskiy settlement, Republic of Karelia, Russia) (Figure 1). Visualization of the geographical location of the sampling area was performed using Google Earth Pro (v.7.3.6.9796). Biosampling for metagenomic analysis was performed on 7 August 2023 in the White Sea bay named “Laminaria dump”, 100 m northeast of the main pier of the White Sea Biological Station, at a depth of 15 m (66°34′16.94″ N, 33°06′23.44″ E), where the seawater temperature was 14 °C.

As a result, two biosamples, designated as Beg5 and Beg14, in the form of thallomes of brown multicellular laminaria algae coated with intense and scant whitish sulfur mats, respectively, were collected from closely located sites of the seabed, placed in 2.5 L vessels, filled to the top with seawater at the collection depth, hermetically sealed with screw caps and lifted from the depth of the White Sea. In the White Sea Biological Station laboratory, the contents of the vessels were poured into 3 L bowls, covered with sheets of paper and left in a dark place at 16–17 °C for same-day analyses. The DNA isolation was also carried out there within two days immediately after sampling. For longer-term operations, during a week, the bioassays were stored at 4 °C.

Physicochemical parameters of the seawater from the sampling sites (pH, temperature and redox potential) were measured with a HI18314F pH meter (Hanna Instruments, Vöhringen, Germany). The concentration of acid-labile sulfide in the samples was determined by the spectrophotometric method with *p*-phenylenediamine and direct iodometric titration, preliminarily fixing sulfide with 10% zinc acetate. The concentration of dissolved oxygen in the medium was determined using a HI 9142 oxygen meter (Romania). Total mineralization was determined by the conductivity method using a Multitest KSL-101 conductometer.

### 3.2. Underwater Photography

Underwater photography of the biosampling points was carried out using a GoPro Hero 7 camera (GoPro, San Mateo, CA, USA).

### 3.3. Microscopy and Microphotography

A binocular microscope Nikon SMZ445 (Nikon, Tokyo, Japan) was used to visually analyze large objects at low magnification. Microscopy analysis of cell morphology of the obtained samples of sulfur mats was performed using a Nikon Eclipse Ei light microscope (Nikon, Tokyo, Japan) equipped with objectives CFI BE2 PLAN 10×/0.25, CFI BE2 PLAN 40×/0.65 and CFI Plan Fluor DLL 100×/1.30 Oil with phase-contrast.

Light microscopy images were acquired using a Leica camera provided on a Huawei P20 smartphone (Huawei, Shenzhen, China), which was mounted in place of the microscope eyepiece. Huawei P20 was equipped with a dual rear camera “Leica summilux h1 1.6/27 asph” with the following characteristics: 12MP (f/1.8 aperture, 1.55 µm pixel size, 1/1.7 sensor size, 960 Slo-Mo recording), 20 MP (monochrome, aperture f/1.6).

### 3.4. Genome DNA Isolation and Purification

The whitish mats covering the thallomes of some brown multicellular laminaria algae were carefully separated from the thallomes, taking care not to disperse the mats and not to trap adjacent silt, and collected into 15 mL vessels using Pasteur glass pipettes. After the mats settled to the bottom of the vessels, the top layer of seawater was decanted and the loose sediment was washed with clean seawater and decanted again. The washed mats were transferred into 1.5 mL Eppendorf tubes and sedimented using ‘Eppendorf MiniSpin plus’ and centrifuged at 5000× *g*, 1 min (Eppendorf AG, Hamburg, Germany). Genome DNA from the resulting washed mats was isolated and purified using Diatom DNA mini Prep 200 kit (Isogen Laboratories, Moscow, Russia) according to the manufacturer’s recommendations with some modifications. Briefly, lysis of the prepared samples in the lysis buffer from the kit was carried out for 45 min at 65 °C in a solid-state thermostat “Thermit” supplied with a built-in timer (DNA-Technology, Moscow, Russia), periodically stirring gently up and down until complete dissolution of mat pieces and clarification of the mixture. The isolated and purified genome DNA was stored at −20 °C for about 2 weeks until sequencing.

### 3.5. Metagenome Sequencing and Assembly of MAGs

Metagenomic DNA isolated from Beg5 and Beg14 sulfur mats was sequenced using the Illumina technique. The libraries for Illumina sequencing were prepared using the NEBNext Ultra II DNA library preparation kit (New England Biolabs, Ipswich, MA, USA). The sequencing of these libraries on an Illumina MiSeq instrument in a paired-end format (2 × 300 nt) produced a total of about 3 Gb and 4.7 Gb of sequences for Beg5 and Beg14 samples, respectively. The low-quality read ends (q = 30) were trimmed using Sickle v.1.33 (https://github.com/najoshi/sickle, accessed on 24 November 2023).

Illumnina reads were assembled into contigs using metaSPAdes v.3.15.4 [35] and MEGAHIT v.1.2.9 [36]. The obtained contigs were binned into MAGs using MaxBin v.2.2.7 [37], CONCOCT v.1.0.0 [38] and MetaBAT v.2.15 [39]. The results of binning were combined and optimized by the DAS Tool v.1.1.4 [40]. The obtained MAGs were taxonomically classified using the Genome Taxonomy Database Toolkit (GTDBTk) v.1.5.0 [41] and the GTDB [17]. CheckM2 v.1.0.1 [42] was used to evaluate the completeness and contamination of obtained MAGs.

Two MAGs were assembled from each metagenome, taxonomically assigned to the candidate genera ‘BB20’ and ‘UBA10656’ of the family *Beggiatoaceae*. MAGs of the same genus from the two metagenomes were identical, indicating the presence of the same two *Beggiatoaceae* genotypes in both analyzed mats. Therefore, different sets of reads were used for the assembly of these MAGs. Using the full set of reads obtained for both samples and the metaSPAdes program, we assembled MAG WS_Bin3 representing the ‘BB20’ lineage. MAG WS_Bin1 of the bacterium of the genus ‘UBA10656’ was assembled using the MEGAHIT program from 35% of reads obtained for the Beg14 mat.

### 3.6. Genome Analysis and Annotation

Gene search and annotation were carried out using the RAST server 2 [43], followed by manual correction of the annotation by comparing the predicted protein sequences with the National Center for Biotechnology Information (NCBI) databases. ANI was calculated using an online resource (https://www.ezbiocloud.net/tools/ani (accessed on 8 February 2024)) based on the OrthoANI algorithm, using USEARCH [44]. AAI between the genomes was determined using the aai.rb script from the enveomics collection [45].

For genome-based phylogenetic analysis, GTDB-Tk v.1.5.0 [41] was used to identify 120 single-copy marker genes in the genomes and to create multiple sequence alignments of concatenated amino acid sequences. The maximum likelihood tree was estimated from the alignment by PhyML v. 3.3 [46] using default parameters (LG amino acid substitution model, 4 substitution rates categories modeled by discrete gamma distribution with estimated shape parameter, branch support values calculated by approximate Bayes method).

## 4. Conclusions

Two MAGs were assembled as a result of the metagenome analysis of bacterial sulfur mats formed on decaying laminaria remnants in the sea bay “Laminaria dump” at a depth of 15–18 m in the White Sea. Based on phylogenetic analysis, one of them, MAG WS_Bin3, was assigned to a new genus and species in the family *Beggiatoaceae* and named ‘*Candidatus* Albibeggiatoa psychrophila’ gen., nov., sp. nov. Accordingly, the candidate genus ‘BB20’ was identified, which includes three species: ‘*Ca*. Albibeggiatoa psychrophila’ sp. nov. WS, ‘*Ca*. Albibeggiatoa’ sp. nov. BB20 and ‘*Ca*. Albibeggiatoa’ sp. nov. NOAA.

The other MAG, namely, WS_Bin1, turned out to belong to the previously described species ‘*Ca*. Parabeggiatoa communis’ within the earlier described genus ‘*Ca*. Parabeggiatoa’. Considering that MAG WS_Bin1 belongs to the genus ‘*Ca*. Parabeggiatoa’ on the basis of 16S rRNA gene homology, the candidate genus ‘UBA10656’ was assigned to the genus ‘*Ca*. Parabeggiatoa’, comprising three new species in addition to the species ‘*Ca*. Parabeggiatoa communis’: ‘*Ca*. Parabeggiatoa’ sp. nov. 1 with MAG B3_G6, ‘*Ca*. Parabeggiatoa’ sp. nov. 2 with MAGs B38_G9 and 4572_84, and ‘*Ca*. Parabeggiatoa’ sp. nov. 3 including MAGs B5_G6, B2_G13 and B37_G9. Filamentous sulfur bacteria referred to the genus ‘*Ca*. Parabeggiatoa’ were also found in the intertidal zone of the White Sea, which once again confirms the wide distribution of these representatives and the diversity of their habitats [19,20,24].

Comparative analysis of genomes revealed the main similarities and differences in the metabolism of the genera ‘*Ca*. Parabeggiatoa’ and ‘*Ca*. Albibeggiatoa’. During heterotrophic growth, the energy metabolism of the two genera is presented by the Krebs cycle. In contrast to representatives of the genus ‘*Ca*. Albibeggiatoa’, species of the genus ‘*Ca*. Parabeggiatoa’ lack genes of the key enzymes of glyoxylate shunt, malate synthetase and isocitrate lyase, which are involved in anabolic and catabolic processes.

The presence of genes for appropriate enzyme systems to oxidize reduced sulfur compounds, hydrogen sulfide and thiosulfate to sulfate and elemental sulfur, which in turn is oxidized by means of the complete rDsr complex, indicates the ability of these bacteria to grow lithoautotrophically, while the complete set of Calvin–Benson–Basham cycle genes implies a capacity for lithoautotrophy.

The main metabolic distinctions between the genera ‘*Ca*. Parabeggiatoa’ and ‘*Ca*. Albibeggiatoa’ are related to the conversion processes of nitrogen compounds. According to the sets of genes, of the two genera, only representatives of ‘*Ca*. Parabeggiatoa’ are capable of denitrification with the formation of gaseous products. At the same time, ‘*Ca*. Parabeggiatoa’ sp. nov. 1, ‘*Ca*. Parabeggiatoa’ sp. nov. 2 and representatives of ‘*Ca*. Albibeggiatoa’ are potentially capable of assimilatory and dissimilatory nitrate reduction. The genes of molecular nitrogen-fixation were detected only in ‘*Ca*. Albibeggiatoa’ sp. nov. BB20 and ‘*Ca*. Albibeggiatoa’ sp. nov. NOAA.

As a result, for the first time, we were able to assemble the full genome sequence of the genus ‘*Ca*. Parabeggiatoa’. Based on both the newly obtained and newly identified genomes, a renewed phylogeny of the family *Beggiatoaceae*, in particular, for the genera ‘*Ca*. Parabeggiatoa’ and ‘*Ca*. Albibeggiatoa’, was developed. In addition, insights into the metabolic potential of the colorless sulfur bacteria of these genera were offered.

The family *Beggiatoaceae* has greatly expanded over the last decade, mainly due to metagenomic analysis. However, the description of new taxa in the family is predominantly based on morphology, while genomic data, in contrast, are devoid of this information. This, in turn, has led to a large number of new unidentified genera, while many of the already described genera are absent from the current taxonomy of the family according to GTDB.

Obtaining pure cultures and their genome sequences could help to make a breakthrough in the taxonomy of the family *Beggiatoaceae*. However, an obstacle to this task is the difficulty of culturing, or even the impossibility of culturing, the vast majority of bacteria of this family. Therefore, at present, only a metagenomic approach along with field observations and microscopy analysis of bacterial morphology will help to solve the problem of the taxonomy of colorless sulfur bacteria of the family *Beggiatoaceae*.

## Figures and Tables

**Table 1:** General characteristics of MAG WS_Bin1 and MAG WS_Bin3, assembled from the metagenome of the bacterial community of sulfur mats in the sea bay “Laminaria dump”, as well as genomes from GeneBank that are phylogenetically related to the obtained MAGs and were included in the analysis.

Genome (MAG)	Genome Assembly	Genome Size (MB)	Contigs	GC-Content (%)	Genes		
					Protein-Coding	16S rRNA	tRNA
*Beggiatoaceae* sp. WS_Bin1	GCA_035871815.1	8.64	1575	41.0	6942	1	45
*Beggiatoaceae* sp. WS_Bin3	GCA_035829175.1	3.64	708	38.0	2800	1	33
MAG B3_G6	GCA_003646175.1	8.8	1343	41.8	6453	0	56
MAG B38_G9	GCA_003646135.1	9.4	888	42.9	6502	0	64
MAG 4572_84	GCA_002085445.1	8.5	406	42.8	6041	0	53
MAG B2_G13	GCA_003645315.1	8.3	1040	41.0	6541	0	43
MAG B37_G9	GCA_003645245.1	8.2	977	40.9	6555	0	47
MAG B5_G6	GCA_003645185.1	8.4	853	40.9	6645	0	40
MAG BB20	GCA_016744725.1	4.5	296	37.9	3939	0	31
*Thiotrichaceae* sp. R2S4C1D_S41.012	GCA_025800385.1	4.8	257	38.5	3584	1	38

**Table 2:** Characterization of the main metabolic pathways of the genera of the family *Beggiatoaceae*. *narG*, membrane-bound nitrate reductase; *nirS*, dissimilatory nitrite reductase; *norBC*, nitric oxide reductase; *nosZ*, nitrous oxide reductase; *napAB*, periplasmic nitrate reductase; *nirBD*, assimilatory nitrite reductase; *nasA*, assimilatory nitrate reductase; *nasD*, assimilatory nitrite reductase; *nif* gene cluster, nitrogenase genes; *sqr*, sulfide:quinone oxidoreductase; *fccAB*, flavocytochrome sulfide dehydrogenase; *soxAXBYZ*, the branched Sox-system; rDsr, complex for sulfur oxidation *dsrABEFHNEMKLJONR*; *soeABC*, quinone-dependent sulfite dehydrogenase; *aprAB*, adenosine 5′-phosphosulfate reductase; *sat*, ATP-sulfurylase, dissimilatory-type. ‘*Ca*. Parabeggiatoa’: MAG WS_Bin1, ‘*Ca*. Parabeggiatoa communis’; MAGs B5_G6, B2_G13, B37_G9, ‘*Ca*. Parabeggiatoa’ sp. nov. 3; MAG B3_G6, ‘*Ca*. Parabeggiatoa’ sp. nov. 1; MAGs B38_G9, 4572_84, ‘*Ca*. Parabeggiatoa’ sp. nov. 2. ‘*Ca*. Albibeggiatoa’: MAG WS, ‘*Ca*. Albibeggiatoa psychrophila’ sp. nov.; MAG BB20, ‘*Ca*. Albibeggiatoa’ sp. nov.; MAG NOAA, ‘*Ca*. Albibeggiatoa’ sp. nov. A plus “+” or minus “−” sign indicates the presence or absence of a metabolic pathway.

Metabolic Pathways				‘*Ca*. Parabeggiatoa’							‘*Ca*. Albibeggiatoa’		
				WS_Bin1	Species 3			Species 1	Species 2		WS	BB20	NOAA
					B5_G6	B2_G13	B37_G9	B3_G6	B38_G9	4572_84			
Nitrogen metabolism	Denitrification, nitrate reductase from the Nar family		NO_3_^−^ → NO^−^_2_	*narGHIJ*	*narGHIJ*			*narGHIJ*	*narGHIJ*		−		
			NO_2_^−^ → NO	−	*nirS*			−	*nirS*				
			NO → N_2_O	*cnorBC*	*cnorBC*			*cnorBC*	*cnorBC*				
			N_2_O → N_2_	−	*nosZ*			−	*nosZ*				
	Dissimilatory reduction of NO_3_^−^ to NH_4_^+^, nitrate reductase from the Nap family			*napAB*	*nirBD*			*napAB*, *nirBD*			*napAB*, *nirBD*		
	Assimilatory reduction of NO_3_^−^ to NH_4_^+^, nitrate reductase from the Nas family			*nasA*, *nasD*							*nasA*, *nasD*		
	Molecular nitrogen fixation			-							-	*nifASUBXX2ENQVWMHDKZTO*	
Dissimilatory sulfur metabolism	Hydrogen sulfide oxidation systems			*sqrAF*, *fccAB*	*sqrAF*, *fccAB*	*sqrAF*, *fccAB*	*sqrA*, *fccAB*	*fccAB*	*sqrAF*, *fccAB*	*sqrAF*, *fccAB*	*sqrF*, *fccAB*		
	Thiosulfate oxidation, Sox-system			*soxAXBYZ*	*soxAXBYZ*	*soxAXYZ*	*soxAXBYZ*	*soxAXBY*	*soxAXBYZ*	*soxBYZ*	*soxAXBYZ*		
	Elemental sulfur oxidation system, rDSR			*dsrABEFHCMKLJOPNRS*									
	Sulfite oxidation systems	Direct way		*soeABC*	*soeABC*	*soeAB*	*soeA*	*soeA*	*soeABC*	*soeABC*	*soeABC*		
		Indirect way		*aprAB*, *sat*	*aprAB*	*aprAB*	*aprAB*, *sat*	*aprAB*, *sat*	*aprAB*, *sat*	*aprAB*	*sat*		
Carbon metabolism	Krebs cycle			+							+		
	Glyoxylate pathway			−							+		
	Pentose-phosphate pathway			+							+		
	Type of RuBisCO			II				IAq			II		

## Data Availability

The original contributions presented in the study are included in the article, further inquiries can be directed to the corresponding authors.
